# How different training sessions affect the sleep of professional soccer players

**DOI:** 10.5114/biolsport.2025.142644

**Published:** 2024-10-15

**Authors:** Eider Barba, David Casamichana, Julen Castellano

**Affiliations:** 1 Real Sociedad Sports Performance Department, Donostia-San Sebastián, Spain; 2 University of the Basque Country (UPV/EHU), GIKAFIT Research Group, Vitoria-Gasteiz, Spain

**Keywords:** Sleep, Football, Monitoring, GPS, Female

## Abstract

This study aimed to compare microcycle loading and sleep patterns concerning successive match days (MD) using objective measurements of sleep and external training load (eTL). Twenty professional female soccer players (mean age: 23.3 ± 3.5 years) were monitored using a multi-sensor sleep-tracker for sleep patterns and global positioning system devices for eTL. Sleep variables included total sleep time (TOTAL), awake time (AWAKE), REM sleep, deep sleep time (DEEP), light sleep time (LIGHT), percentage of sleep time spent moving (REST), and sleep onset latency (ONSET). eTL variables included total distance covered (TD), distance at various speed thresholds, accelerations (ACC3), and decelerations (DEC3). 1) eTL influenced sleep; 2) TOTAL and REM were reduced after high eTL sessions (MD-3); 3) MD-2 facilitated recovery and improved post-session sleep compared to MD-4 and MD-3; 4) post-session TOTAL and DEEP sleep times on MD-1 were shorter compared to pre-session times, but there was no difference in REM sleep. Additionally, DEEP had a negative correlation with eTL variables, while REST had a positive correlation with certain eTL variables. REM sleep was affected after high eTL sessions, indicating that fatigue negatively impacted sleep. However, sleep increased after lower eTL sessions. Players slept less with less DEEP sleep the day before a competition (MD-1 post-session sleep). Monitoring sleep during microcycle acquisition and loading phases could assess sleep changes. Strategies should be implemented to improve sleep during loading phases and the night before matches.

## INTRODUCTION

Sleep is a crucial biological necessity for human well-being and health [1]. During periods of sleep restriction or deprivation, overall health is negatively affected, particularly the immune system [2], endocrine system [2], risk of injury [3], physical [4], mood [4] or cognitive functions [4]. In the context of sports, sleep provides several essential psychological and physiological functions that may be fundamental to the recovery process [5], so the need for adequate sleep has become one of the fundamental pillars of recovery from the fatigue created by training and competition [6, 7]. Given the growing concern about athletes’ and soccer players’ sleep, studies examining sleep interventions have grown exponentially over recent years [8].

Advances in technology to measure the quantity and quality of sleep (e.g., rings, bracelets, watches) place practitioners in a sustainable context to monitor sleep data systematically. This method has a higher level of accuracy than when players are asked to fill out self-reported questionnaires on sleep durations as a control tool [9]. The gold standard technology for measuring sleep is polysomnography (PSG). However, this technology is expensive and less convenient than others [10], so its use is often limited to a few sessions or weeks of monitored use [11]. Good alternative methods to PSG are wearable technological devices, such as actigraphy, smart watches, smart rings, wristbands, or fitness trackers [12, 13]. So far, the most widely used technology has been actigraphy, because it is a validated and reliable technology that is simple and easy to use daily [10]. However, other technologies have been gaining momentum as they allow devices to automatically detect sleep in real time, providing sleep metrics the next morning through a digital platform (e.g., a smartphone app), which makes them easy to use in everyday life [10, 13]. However, the considerable variation between these commercial devices should be noted [13].

In soccer players, sleep may be related to decreased athletic performance [14]. A night of poor sleep is associated with an increase in reaction times and a decrease in cognitive processes such as visual tracking, concentration, determination, and mood [15]. Moreover, it is also related to injury risk [14]. Several studies have found that soccer players with shorter sleep duration and poor sleep quality increased the number and severity of musculoskeletal injuries [3, 16]. The causes of these sleep disturbances in soccer players are divided into two groups: sport-specific factors and non-sport factors [17]. Sport-specific factors are associated with trans-meridian travel (disrupting circadian rhythm), an unfamiliar sleep environment, cognitive arousal the night before a competition, training/competition stress, evening competitions, high training/competition loads, and early morning training [17]. Non-sport factors are related to social life, work/student commitments, lifestyle, individual characteristics, attitudes and beliefs, and family commitments [17].

If we focus on the external training/match load factor, many studies indicate that periods of workload intensification increase the level of disturbances associated with sleep outcomes [18, 19]. Disturbances could be the result of overstrain and proinflammatory responses [18, 19]. Clemente et al. [20] suggested that congested periods involving increased stress and stiffness are more likely to affect sleep than regular periods. Even so, these studies have several limitations, since the data were collected in a tournament for only five [19] and nine days [18]. In the case of Figueiredo et al. [19], the study was performed on non-professional male players under 15 years old.

At the professional level, training periodization is a relevant aspect of contemplating adequate load management [21]. An inverted U-shaped periodization is usually proposed [22], where the central days of the microcycle are used to develop the conditional aspects or acquisition days in the tactical periodization [23]. It is usually proposed that each training session in the competitive microcycle has a priority conditional objective, trying to avoid coinciding on successive days to avoid overloading the same conditional dimension [22]. To the authors’ knowledge, it is not yet known how it affects sleep in each training session of a competitive microcycle, which would favour the balance between work and rest necessary for the player to be able to respond and adapt to the loads that are demanded of him/her. However, there needs to be more objectively measured information on sleep and external training load (eTL). To our knowledge, research studies have yet to take into account the weekly microcycle to analyse eTL and sleep. Therefore, this study aimed to 1) compare microcycle eTL and sleep patterns as a function of match day (MD) in female soccer players using objective data to measure both sleep and eTL and 2) analyse how eTL affects the sleep, and vice versa, of female soccer professional players in different types of training sessions in a competitive microcycle.

## MATERIALS AND METHODS

### Subjects

Twenty professional female players (age: 23.3 ± 3.5 years; height: 169.7 ± 5.5 cm; body mass: 64.6 ± 5.3 kg) who played in the Spanish Women’s First Division (*Liga Profesional Femenina*) participated in this study. The inclusion criteria in the study were as follows: 1) Only players who participated in training sessions and submitted sleep records were included in the analysis; if they did not have one of the two records, that day was not considered for the analysis; 2) goalkeepers were excluded from the analyses due to their specific role.

The GPS data arose as a condition of the players’ employment whereby they were assessed daily; thus, no authorization was required from an institutional ethics committee [24, 25]. Instead, for the collection of ŌURA data, the authorization of an institutional ethics committee was required (C2TI0327). All players who participated in the study signed a document agreeing to the use of the ŌURA ring device. This study conformed to the Declaration of Helsinki and players provided informed consent before participating, and the identities of the players were anonymized.

### Measures

The eTL was collected using GPS devices (Realtrack Systems SL, Almeria, Spain). Players wore the GPS devices from the beginning of the session until the end. The GPS device was fitted to the upper back (i.e., between the shoulder blades) of each player in a specifically designed neoprene harness to minimise movement artefacts [26]. After each session, the data were extracted to a computer and analysed using SPRO (V.990) of WIMU. The eTL variables analysed were similar to those used in previous studies on professional female soccer players [27]: total distance covered (TD, m), distance at > 14 km · h^−1^ (TD14, m), distance at > 18 km · h^−1^ (TD18, m), distance at > 21 km · h^−1^ (TD21, m), distance at > 24 km · h^−1^ (TD24, m), number of accelerations > 3 m · s^−2^ (ACC3, n) and decelerations > 3 m · s^−2^ (DEC3, n).

Sleep data were collected using a multi-sensor sleep-tracker (ŌURA ring, Gen2). The ŌURA ring employs an array of sensors—motion, body temperature, resting heart rate, heart rate variability, respiratory rate and pulse wave variability amplitude—to monitor sleep and determine sleep stages [28]. The players wore the ring throughout the day except during training sessions and matches, as the regulations do not allow them to wear rings [29]. Rings are waterproof, made in ceramic, come in different sizes (U.S. standard ring sizes 6–13) and weigh about 15 g, with a battery life of about three days. The ring automatically connects via Bluetooth and transfers data to a mobile platform via the app Movile. The sleep variables analysed were the following: total duration of sleep (TOTAL, in min), total duration of awake time (AWAKE, in min), total duration of REM sleep (REM, in min), deep sleep time (DEEP, in min) and light sleep time (LIGHT, in min), percentage of sleep time spent moving (REST, %) and the detected latency from bed time to the beginning of the first five minutes of persistent sleep (ONSET, in min). All these variables were measured both before and after a session. The difference between the records of one day’s post-session sleep and the next day’s pre-session sleep, despite being the same night, was the number of records used. If a player participated in one session but did not participate in the next, her record was counted for the post-session sleep after the first session but not for the pre-session sleep before the second session.

### Procedures

The study was conducted during the seasons 2021–2022 and 2022–2023. A total of 10 microcycles were analysed during the competitive period: five microcycles of the 2021–2022 season and five microcycles of the 2022–2023 season. The other microcycles of the season did not meet the established requirements. To categorize the training sessions, the days-to-match approach was used [23]. MD-4: The session was performed four days before the next match and was force/power development oriented. These sessions were dominated by small-sided games with goalkeepers in reduced areas per player (< 50 m^2^). MD-3: performed three days before the next match and aiming at the development/maintenance of endurance but with the tactical-related drill as the main content. These sessions were programmed with large-sided games and large areas per player (250–300 m^2^). MD-2: The training session took place two days before the next match and involved speed development orientation in situations close to competition. In these sessions, the activity was intermittent, avoiding fatigue in the players. MD-1: The pre-competition session, taking place one day before the match, aimed to perform tapering mainly focused on activation exercises that reproduced the tactical scenarios of the competition and concluded with set pieces.

A total of 253 individual files were analysed, with the following distribution: MD-4 = 47, MD-3 = 68, MD-2 = 76, and MD-1 = 62 files. The average number of registrations per player was 18.0 ± 14.1 files (minimum: 2 and maximum: 50 individual files). For the analysis, we selected microcycles with the same structure: previous MD and two first days MD+1 and day-OFF (not included in the study), followed by MD-4, MD-3, MD-2, MD-1 and MD (not included in the study). We omitted microcycles with two matches or another periodization mentioned above in the analysis.

### Statistical analysis

The descriptive statistics were calculated and reported as mean ± standard deviation of the mean (SD) for each training session relative to MD, for each variable. For eTL analysis, the differences between the independent variable MD (−) training session in all measured variables (dependent variables) were examined using repeated measures analysis of variance (ANOVA). To analyse sleep, differences between the independent variables MD (-) training session and pre-postsession values in all measured variables (dependent variables) were examined by repeated measures analysis of variance (ANOVA). Post hoc analyses were performed using Bonferroni’s honestly significant difference test. Differences between training sessions were assessed via standardized mean differences (Cohen’s d with 95% confidence limits). The interpretation thresholds for standardized effect size (ES) were as follows [30]: < 0.2 (trivial), 0.2–0.6 (small), 0.6–1.2 (moderate), 1.2–2.0 (large), and > 2.0 (very large). Spearman’s rho correlation analysis was used to assess bivariate relationships [31] with coefficients categorised as r ≤ 0.4 (weak), r = 0.4–0.6 (moderate), r = 0.6–0.8 (strong) or r = 0.8–1.0 (very strong). JASP version 0.18.1 was used to conduct the analysis and statistical significance was set at p < 0.05. In the results section, three significance levels have been differentiated: p < 0.05, p < 0.01 and p < 0.001.

## RESULTS

Table 1 presents the absolute eTL variables obtained in training sessions according to the days before the MD. Regarding global indicators, MD-3 displayed higher values than MD-4, MD-2 and MD-1 in TD, TD14, TD18, TD21 and TD24 (p < 0.001; ES ranged from 0.80 to 2.87) and than MD-2 in ACC3 and DEC3 (p < 0.01; ES = 0.43–0.63). MD-1 showed the lowest eTL in TD, TD14, ACC3 and DEC3 (p < 0.05; ES = 0.54–2.87) concerning the other training sessions and lower eTL in TD18, TD21 and TD24 with respect to MD-2 and MD-3 (p < 0.01; ES = 0.52–2.24), with no significant differences between MD-1 and MD-4 in these last eTL variables. Finally, MD-4 accumulated more ACC3 and DEC3 than the other training sessions (p < 0.001; ES = 0.67–2.87) and less distance covered in TD18, TD21 and TD24 than MD-2 (p < 0.01; ES = 0.45–0.66).

**TABLE 1:** Absolute external training load variables for training sessions according to type of day.

Variables	MD-4	MD-3	MD-2	MD-1
TD (m)	4990.9 (678.2)	6318.7 (1219.1)	4719.9 (972.8)	3744.8 (503.5)

ES (95% CI)	MD-4 > MD-1 (1.39, 0.9/1.9)	MD-3 > MD-4 (1.48, -1.9/-1.0) MD-3 > MD-2 (1.78, 1.3/2.2) MD-3 > MD-1 (2.87, 2.3/3.4)	MD-2 > MD-1 (1.09, 0.6/1.5)	

TD14 (m)	457.5 (188.3)	939.4 (385.7)	549.7 (222.4)	320.2 (126.1)

ES (95% CI)	MD-4 > MD-1 (0.54, 0.1/1.0)	MD-3 > MD-4 (1.90, -2.4/-1.4) MD-3 > MD-2 (1.54, 1.1/2.0) MD-3 > MD-1 (2.45, 1.9/2.9)	MD-2 > MD-1 (0.91, 0.5/1.3)

TD18 (m)	107.1 (67.0)	336.0 (168.7)	182.2 (116.0)	81.1 (63.6)

ES (95% CI)		MD-3 > MD-4 (2.01, -2.5/-1.5) MD-3 > MD-2 (1.35, 0.9/1.8) MD-3 > MD-1 (2.24, 1.8/2.7)	MD-2 > MD-1 (0.89, 0.5/1.3 )MD-2 > MD-4 (0.66, -1.1/-0.2)	

TD21 (m)	37.5 (58.1)	169.4 (116.8)	82.7 (73.3)	28.4 (40.1)

ES (95% CI)		MD-3 > MD-4 (1.68, -2.2/-1.2) MD-3 > MD-2 (1.11, 0.7/1.5) MD-3 > MD-1 (1.80, 1.3/2.3)	MD-2 > MD-4 (0.58, -1.0/-0.1) MD-2 > MD-1 (0.69, 0.3/1.91)	

TD24 (m)	7.4 (23.4)	55.9 (61.5)	54.0 (35.00)	4.9 (16.4)

ES (95% CI)		MD-3 > MD-4 (1.25, -1.7/-0.8) MD-3 > MD-2 (0.80, 0.4/1.2) MD-3 > MD-1 (1.32, 0.9/1.8)	MD-2 > MD-4 (0.45, -0.9/0.0) MD-2 > MD-1 (0.52, 0.1/0.9)	

ACC3 (n)	37.5 (12.4)	26.8 (8.8)	22.9 (7.4)	17.7 (7.0)

ES (95% CI)	MD-4 > MD-3 (1.18, 0.7/1.6) MD-4 > MD-2 (1.62, 1.1/2.1) MD-4 > MD-1 (2.19, 1.7/2.7)	MD-3 > MD-2 (0.43, 0.0/0.9) MD-3 > MD-1 (1.00, 0.6/1.4)	MD-2 > MD-1 (0.57, 0.1/1.0)	

DEC3 (n)	46.3 (14.1)	38.6 (12.0)	31.2 (11.3)	23.3 (8.4)

ES (95% CI)	MD-4 > MD-3 (0.67, 0.2/1.1) MD-4 > MD-2 (1.31, 0.9/1.8) MD-4 > MD-1 (1.99, 1.5/2.5)	MD-3 > MD-2 (0.64, 0.2/1.1) MD-3 > MD-1 (1.32, 0.9/1.8)	MD-2 > MD-1 (0.69, 0.3/1.2)	

Note: MD is match day; TD is total distance covered; TD14 is distance covered at > 14 km · h^−1^; TD18 is distance covered at > 18 km · h^−1^; TD21 is distance covered at > 21 km · h^−1^; TD24 is distance covered at > 24 km · h^−1^; ACC3 is number of accelerations> 3 m · s^−2^; DEC3 is number of decelerations > 3 m · s^−2^. ES is the effect size and the 95% confidence interval (CI) of the mean is a range with an upper and lower number calculated from a sample. ES (95% CI) is only shown for significant differences (p < 0.05).

Table 2 shows the pre-session and post-session sleep variables according to the days before the MD. There were significant differences between pre-session and post-session TOTAL on MD-3, MD-2 and MD-1 (p < 0.001; ES = 0.47–0.94). MD-3 and MD-1 presented higher pre-session values and MD-2 higher post-session values. The pre-session MD-3 REM value was higher than the post-session value (p < 0.05; ES = 0.41). Finally, the pre-session MD-1 had a higher DEEP value compared to the post-session value (p < 0.01; ES = 0.60). None of the days presented significant differences between before and after the session in the variables AWAKE, LIGHT, REST and ONSET (p > 0.05; ES = 0.00–0.38).

**TABLE 2:** Pre- and post-session sleep variables according to the number of days before the match day.

Sleep	MD-4		MD-3		MD-2		MD-1	
Variables	Pre-S	Post-S	Pre-S	Post-S	Pre-S	Post-S	Pre-S	Post-S
TOTAL (min)	447.1 (49.0)	449.1 (47.3)	448.8 (45.7)	424.2 (50.5)	424.9 (51.2)	458.2 (48.9)	457.6 (52.9)	408.68 (76.3)

ES (95% CI)	MD-4 > MD-1 (0.73, -1.3/-0.2)*		Pre > Post (0.47, 0.0/0.9)		Post > Pre (0.64, -1.1/-0.2) MD-2 > MD-1 (0.89, -1.4/-0.4)* MD-2 > MD-3 (0.62, 0.2/1.1)*		Pre > Post (0.94, 0.4/1.5)	

AWAKE (min)	68.5 (24.0)	70.3 (24.6)	79.3 (25.9)	60.8 (23.9)	62.5 (26.7)	72.5 (32.6)	73.5 (34.7)	67.3 (28.1)

ES (95% CI)								

REM (min)	68.3 (28.7)	71.4 (30.7)	73.0 (30.2)	61.7 (25.9)	61.7 (26.3)	69.3 (28.0)	69.5 (21.1)	61.6 (24.4)

ES (95% CI)			Pre > Post (0.41, 0.0/0.8)					

DEEP (min)	123.9 (29.6)	123.0 (30.9)	127.2 (34.8)	128.9 (35.3)	128.2 (34.2)	133.9 (32.3)	135.0 (35.2)	115.3 (31.3)

ES (95% CI)					MD-2 > MD-1 (0.57, -1.1/-0.6)*		Pre > Post (0.60, 0.1/1.1)	

LIGHT (min)	255.3 (51.9)	255.1 (57.5)	249.0 (53.1)	234.0 (60.2)	235.4 (56.6)	255.4 (53.5)	253.5 (54.9)	232.2 (62.1)

ES (95% CI)								

REST (min)	8.6 (8.2)	8.8 (8.7)	8.9 (8.8)	8.7 (8.5)	8.5 (8.4)	8.3 (8.4)	7.6 (7.5)	7.9 (6.9)

ES (95% CI)								

ONSET (min)	16.0 (12.4)	13.6 (7.2)	13.4 (7.8)	12.6 (8.7)	13.3 (8.9)	12.4 (9.3)	11.6 (8.7)	14.9 (9.5)

ES (95% CI)								

Note: Pre-S is pre-session; Post-S is post-session; MD is match day; TOTAL is total duration of sleep; AWAKE is total duration of awake time; REM is total duration of REM sleep; DEEP is total duration of deep sleep; LIGHT is total duration of light sleep; REST is the percentage of sleep time spent moving; ONSET is the latency from bed time to the beginning of the first five minutes of persistent sleep. ES is the effect size and the 95% confidence interval (CI) of the mean is a range with an upper and lower number calculated from a sample. ES (95% CI) is only shown for significant differences (p < 0.05).

* Differences between days according to match day have been calculated using Post-S data.

On the other hand, there were significant differences between the different days of the microcycle, according to MD. The day with the highest post-session TOTAL value was MD-2, with a significant difference between MD-1 and MD-3 post-session values (p < 0.05; ES = 0.62–0.89). On MD-4 post-session TOTAL was also longer than on MD-1 (p < 0.05; ES = 0.73). The other significant difference observed was in the time in DEEP, with more time on MD-2 after the session than on MD-1 (p < 0.05; ES = 0.57).

**FIG. 1:**
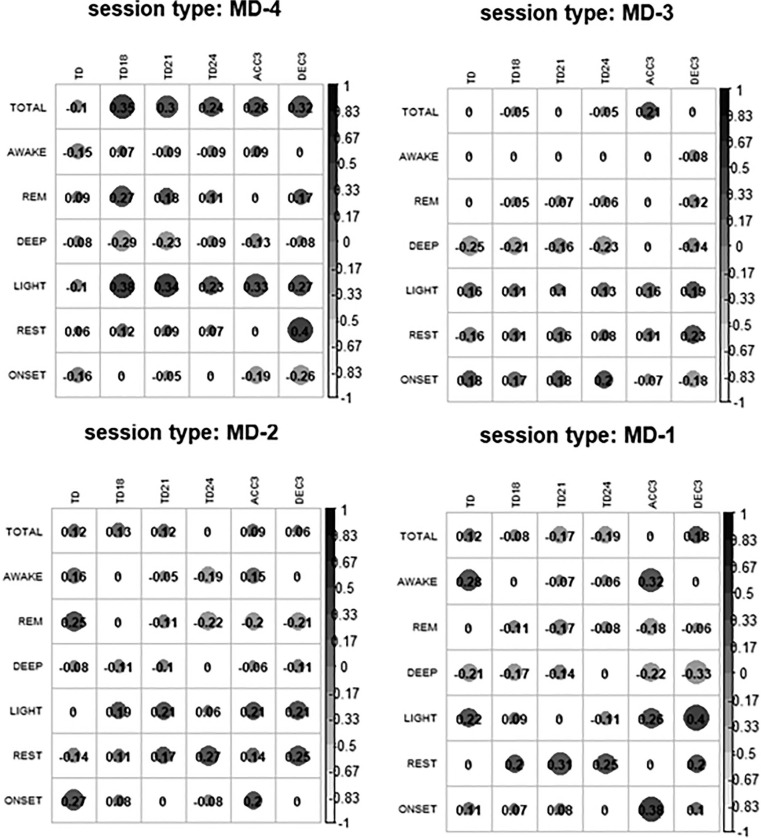
Correlations between external training load variables and pre-session sleep. Note: MD is match day; TOTAL is total duration of sleep; AWAKE is total duration of awake time; REM is total duration of REM sleep; DEEP is total duration of deep sleep; LIGHT is total duration of light sleep; REST is the percentage of sleep time spent moving; ONSET is the latency from bed time to the beginning of the first five minutes of persistent sleep; TD is total distance covered; TD14 is distance covered at > 14 km · h^−1^; TD18 is distance covered at > 18 km · h^−1^; TD21 is distance covered at > 21 km · h^−1^; TD24 is distance covered at > 24 km · h^−1^; ACC3 is number of accelerations > 3 m · s^−2^; DEC3 is number of decelerations > 3 m · s^−2^. All correlations that are not 0 are significant correlations.

The correlations between the pre-session sleep variables and the eTL variables were null or very small for most of the relationships in each type of session studied (Figure 1). On MD-4 there was a correlation of TOTAL and LIGHT with the eTL variables, and on MD-3 there was a negative correlation between eTL and DEEP. On MD-2 we observed that REM sleep went from having a null or small positive correlation to a small negative correlation with TD24, ACC3 and DEC3. REST and LIGHT were also correlated with eTL. Finally, on MD-1 DEEP continued to have a negative correlation with eTL and the correlations of rest with TD18, TD21, TD24 and LIGHT with ACC3 and DEC3 increased.

**FIG. 2:**
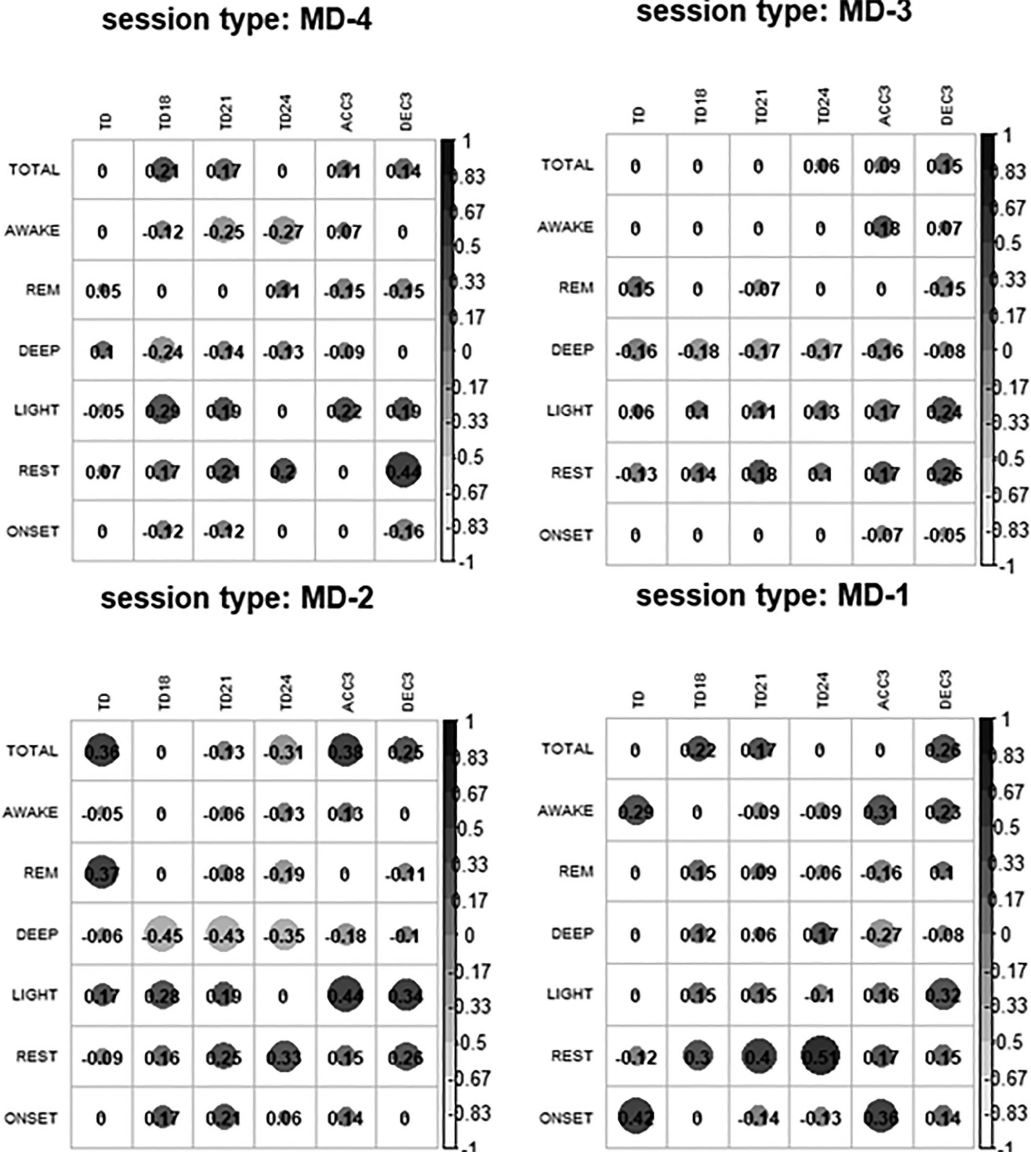
Correlations between external training load and post-session sleep variables. Note: MD is match day; TOTAL is total duration of sleep; AWAKE is total duration of awake time; REM is total duration of REM sleep; DEEP is total duration of deep sleep; LIGHT is total duration of light sleep; REST is the percentage of sleep time spent moving; ONSET is the latency from bed time to the beginning of the first five minutes of persistent sleep; TD is total distance covered; TD14 is distance covered at > 14 km · h^−1^; TD18 is distance covered at > 18 km · h^−1^; TD21 is distance covered at > 21 km · h^−1^; TD24 is distance covered at > 24 km · h^−1^; ACC3 is number of accelerations > 3 m · s^−2^; DEC3 is number of decelerations > 3 m · s^−2^.

Similarly, as shown in Figure 2, the eTL variables had a null or very small correlation with the post-sleep variables for most of the relationships in each type of session. In MD-4, the negative correlations of AWAKE and DEEP with TD18, TD21, TD24 and the positive correlations between LIGHT and REST with eTL stand out. On MD-3 there was a negative correlation between DEEP and eTL and a positive correlation between LIGHT and REST with ACC3 and DEC3. On MD-2 the same occurred as on MD-3 but the correlations were even stronger. Finally, on MD-1 there was a correlation between REST and TD18, TD21 and TD24.

## DISCUSSION

This study aimed to compare microcycle eTL and sleep patterns as a function of MD in female soccer players and analyse how eTL affects the sleep and vice versa of female soccer professional players in different types of training sessions in a competitive microcycle. The results for the first objective revealed that: 1) a different eTL affected the players’ sleep; 2) the variables TOTAL and REM were affected (reduced) after the session with the highest locomotor stimulation of the microcycle (MD-3); 3) although the sleep got worse with the accumulation of the eTL of the MD-4 plus MD-3 sessions, the MD-2 session fulfilled the objective of facilitating recovery and improving the post-session sleep, being the day with the longest sleep time of the entire microcycle; 4) the post-session MD-1 (night before the match) TOTAL variable and DEEP sleep were shorter compared to the pre-session values; even so, there were no differences in REM sleep. On the other hand, the results for the second objective revealed that: 5) the DEEP variable was negatively correlated with the eTL variables (higher eTL, less DEEP sleep) and the REST variable was positively correlated with TD18, TD21 and TD24 on post-session MD-1 (higher eTL of these variables on MD-1, more movement during sleep). The main conclusion of the study was that the eTL or the proximity to the official match influenced the sleep of the players.

To the authors’ knowledge, this is the first study in which female professional soccer players have been studied comparing the sleep measured by microelectromechanical system (MEMS) devices throughout the training sessions that make up a typical in-season microcycle (e.g., four consecutive training sessions: MD-4, MD-3, MD-2 and MD-1 after 3 days of the previous match and with an off day on the second day), all with objective measurements of both sleep and eTL. The literature about the eTL has tended to report that the MD-4 session is used for giving neuromuscular priority (ACC3 and DEC3) with respect to the other training session and the MD-3 session for giving prominence to locomotor stimulation (higher values in all the displacement variables: TD, TD14, TD18, TD21 and TD24) [22, 23]. In the present study, an eTL profile was described that followed the tendencies of the literature. On the other hand, until now it had been concluded that eTL negatively affects sleep in congested periods [18] and in tournaments [19]. In this study we observed that a different eTL affected the players’ sleep, with TOTAL and the REM sleep being impaired especially after one the session with the highest eTL of the microcycle (MD-3), being the last day of the phase with the most eTL of the microcycle (MD-4 and MD-3 as the acquisition phase of the microcycle). Therefore, in this study we observed that two consecutive training sessions of intense training (MD-4 plus MD-3 sessions) is enough to negatively affect sleep.

The tapering strategy has proven to be effective in increasing match performance [32]. This decrease in eTL as the match approaches seems to be a strategy frequently adopted by soccer teams, with the intention of favouring recovery processes before the match [22, 32, 33]. In this study we observed that the tapering strategy was effective in the players’ sleep, concluding that the MD-2 session fulfilled the objective of facilitating recovery, through the reduction of the eTL and achieving an increase in the amount of sleep time.

Previous studies showed that athletes tend to sleep worse the day before a competition [34, 35]. There are several factors that alter the total sleep of players on MD-1 [17], such as pre-competition stress, travel, sleeping away from home or the competition schedule. In our study, despite a reduction in the eTL, the MD-1 post-session DEEP sleep, the night before the match, was shorter; even so, there were no differences in restorative sleep. However, despite sleeping worse, the REM phase of sleep in our study was not affected; differences were only observed in the DEEP sleep phase. We observed that eTL may be another factor affecting MD-1 players’ sleep, as high eTL in TD18, TD21 and TD24 variables was related to having more LIGHT sleep and more movement during sleep (REST).

Variations in the perceived fatigue of the players and sleep have also been studied [36], showing that both the REM and DEEP phases are associated with fatigue. Based on the analysis of the correlations, most of the variables presented a null or very small correlation with eTL. The most notable correlation was the negative correlation between the DEEP phase of sleep and general eTL. In relation to previous findings, our study confirmed this idea with objective data by finding this negative correlation; that is, the higher the accumulated weekly eTL was, the shorter was the DEEP sleep.

This research study has limitations. Firstly, it should be mentioned that, as is usual in this type of study, the results are limited to a single professional team, which should be considered when attempting to generalize the results to other teams or areas of application. A second limitation of the study is that it was only possible to analyse a standard competitive microcycle with four training sessions prior to the official match. However, it is usual that throughout the season, there are microcycles with a different number of days between matches or microcycles with two competitions. Furthermore, information has yet to be provided on the day of the match and the two days following the match, which could provide information relevant to the study. Another limitation of the study is that the postmatch MD-1 sleep was analysed without considering whether the players slept at home or in a hotel after travelling for the match. Finally, sleep can be affected by several factors beyond our control.

## CONCLUSIONS

The study’s main conclusions are that the REM phase was affected after the highest eTL sessions of the microcycle, concluding that the fatigue provoked on MD-4 and MD-3 negatively affected sleep. However, the amount of sleep increased after the MD-2 training session, with lower eTL than in previous sessions. Finally, players slept less and with less time in the DEEP phase of sleep the day before the competition (MD-1 post-session sleep).

The application relevance of the study was that sleep could be affected by eTL, so it may be interesting to monitor sleep in the acquisition phase of the microcycle to assess the degree of decrease of sleep after the loading phase of the microcycle to find out if sleep increases, as well as the night before the match to determine how it is affecting the players. On the other hand, strategies should be established to improve sleep in the loading phases of the microcycle as well as on the night before the match.
